# Characterization of a novel panel of polymorphic microsatellite loci and the mitogenome for the Neotropical marsupial *Gracilinanus agilis* (Didelphimorphia: Didelphidae)

**DOI:** 10.1007/s11033-026-11765-3

**Published:** 2026-04-10

**Authors:** Marcela Reis Duarte da Silva, Cássia Alves Lima-Rezende, Priscilla Lóra Zangrandi, André Faria Mendonça, Renato Caparroz, Emerson Monteiro Vieira, Fernando Pacheco Rodrigues

**Affiliations:** 1 Laboratório de Genética e Biodiversidade, Departamento de Genética e Morfologia, Instituto de Ciências Biológicas, Universidade de Brasília, Campus Universitário Darcy Ribeiro, Brasília, CEP 70910-900 DF Brazil; 2 Programa de Pós-Graduação em Ciências Ambientais, Universidade Comunitária da Região de Chapecó (Unochapecó), Chapecó, CEP 89809-900 SC Brazil; 3 Laboratório de Ecologia de Vertebrados, Departamento de Ecologia, Instituto de Ciências Biológicas, Universidade de Brasília, Campus Universitário Darcy Ribeiro, Brasília, CEP 70910-900 DF Brazil; 4 Laboratório de Ecologia e Conservação Animal, Departamento de Ecologia, Instituto de Ciências Biológicas, Universidade de Brasília, Campus Universitário Darcy Ribeiro, Brasília, CEP 70910-900 DF Brazil

**Keywords:** Genetic parentage, High-throughput sequencing, Mouse opossum, Population genetics

## Abstract

**Background:**

Microsatellite and mitochondrial markers are important tools for investigating natural populations, enabling the assessment of their diversity, genetic structure, kinship, parentage, and reproductive biology. Although the importance of such markers is widely recognized, their use in Neotropical mammals, especially marsupials, is still limited by the scarcity of species-specific loci characterized for extant species.

**Methods and results:**

In this study, we describe and characterize 15 polymorphic microsatellite loci and recover part of the mitochondrial genome for the Neotropical marsupial *Gracilinanus agilis* using massive parallel sequencing with the Illumina platform. DNA sequencing yielded a dataset comprising 2,331,566 paired reads, of which 1,434 were mapped to a previously published mitogenome of the species, resulting in a partial mitogenome of 16,098 base pairs. The microsatellite search identified a total of 4,199 loci, of which 26 pairs were tested on 30 individuals. Of these, 15 loci were successfully amplified and exhibited high levels of genetic variation, with an average of 7.73 alleles per locus, a mean observed heterozygosity of 0.63, and a mean expected heterozygosity of 0.72. Furthermore, these loci collectively provide a high paternity exclusion probability and a low identity probability.

**Conclusions:**

Our results suggest that the described marker set could be very useful for population genetic studies, evaluation of reproductive strategies (such as semelparity), and analysis of the population dynamics of this species.

## Introduction

Microsatellite and mitochondrial markers are important tools for investigating natural populations, enabling the assessment of their diversity, genetic structure, kinship, parentage, and reproductive biology [1, 2]. These tools can unravel previously unknown patterns related to crucial reproductive aspects of mammals, including mating systems and individual contribution to the genetic pool of the next generation [3, 4].

Although this is a well-established tool, in many cases the use of microsatellites depends on the availability of species-specific loci. For Neotropical marsupials, for instance, there are at least 137 known species [5], but microsatellite loci have been described for only 11 of them (see compilation at [6], plus [7, 8]). Similarly, mitogenome sequences are available for only 17 Didelphimorphia species in GenBank. This lack of information hinders the adequate evaluation of relevant aspects of natural history and population trends for most species in this group.

One of the neotropical marsupials with no information on microsatellite markers is the gracile mouse opossum *Gracilinanus agilis* (Burmeister 1854). This small (body weight range from 18 to 43 g) marsupial species occurs mainly in forested habitats in Brazil, eastern Paraguay, Peru and Bolivia [9]. It is an insectivore-omnivore marsupial that feeds on ants, beetles, termites, and fruits [10], making it a potential agent for biological control in agricultural landscapes [11] and an important seed-dispersing agent [12]. In addition, it serves as a food item for other species [13] and is considered a wild reservoir for several pathogens of interest in public and veterinary health [14]. Although *G. agilis* has a wide distribution and is present in several protected areas, where it presumably has large populations, little is known about the current population trends [15]. The continuous decline of forest areas throughout its geographic distribution [16] may lead to a change in its conservation status, which is currently considered to be of *Least Concern* by the International Union for Conservation of Nature (IUCN) [15].

An interesting biological characteristic of this species is its reproductive strategy. Several studies suggest that *G. agilis* is a semelparous species [17–19]. This reproductive strategy (semelparity) is characterized by male mortality and a sharp decline in female fecundity, or even death, after the first breeding season [19]. Although there is no consensus on the magnitude of semelparity in this species, the complete mortality of individuals of both sexes after the breeding season has already been observed in American semelparous marsupials of the genera *Marmosops* and *Monodelphis* [20, 21], resulting in non-overlapping discrete generations.

To date, all population and demographic studies related to *G. agilis* have been performed using the capture-mark-recapture methodology (e.g. [18]). An alternative that can expand our understanding of the magnitude of semelparity in this species is to carry out population studies using microsatellite markers. The use of these markers in parentage and kinship analyses has the potential to provide a more accurate assessment of the reproductive strategy in *G. agilis*, in addition to allowing an evaluation of the diversity and genetic structure of their populations. Here, we present the first polymorphic microsatellite loci isolated and characterized for *G. agilis* using Illumina next-generation sequencing (NGS) data and discuss the usefulness of these markers for population and parentage analyses. Moreover, from the datasets generated by NGS, we also described the mitogenome of *G. agilis*, another important marker for population genetics studies.

## Materials and methods

### Sampling and DNA extraction

Tissue samples were obtained by ear clipping from 30 individuals captured during a long-term ecological study at the Botanical Garden of Brasília and stored in absolute alcohol at -20 °C until use. For next-generation sequencing, genomic DNA from one female individual was extracted using the DNeasy Blood and Tissue Kit (Qiagen), following the manufacturer’s instructions. Genomic DNA from the 29 remaining samples was isolated using a phenol-chloroform protocol based on the method described by [22].

### Genomic library preparation and sequencing

Approximately 1.0 µg of genomic DNA was used for genomic library preparation using a TruSeq Nano DNA HT Sample Preparation Kit (Illumina, USA). The 250 bases of the paired-end reads present in the genomic library were sequenced using the HiSeq 2500 Illumina Platform (Illumina, San Diego, CA, USA). Genomic library construction and sequencing were performed at the GenOne Biotechnologies Facility (http://www.genone.com.br).

### Mitogenome assembly and annotation

Merged and unmerged reads were aligned to *the Gracilinanus agilis* mitogenome reference (GenBank Accession No. NC_054268.1) using GENEIOUS 6.1.8, whereas annotation was performed using both GENEIOUS and the web-based platform MITOS [23]. Necessary adjustments were made manually by comparison with the reference mitogenome using GENEIOUS software.

### Microsatellite search and primer design

Microsatellite loci were identified from raw Illumina paired-end data using a Galaxy-based bioinformatics pipeline [24], which incorporates existing computer programs and filtering scripts. Briefly, (i) the quality of the raw reads was assessed using FastQC v.0.11.4 [25]; (ii) low-quality reads and adapter sequences were removed from the dataset using Trimmomatic v.0.32 [26]; (iii) filtered reads were then inspected to identify microsatellites and potential amplifiable loci in Pal_finder v.0.02.04 [27] with options selected to search perfect di- to hexanucleotide microsatellite loci with a minimum of six repeats; (iv) primer design was carried out using Primer3 [28] with an optimum primer length of 20 bp (18–25 bp), GC content ranging from 30% to 80%, melting temperature ranging from 59 °C to 61 °C, optimum melting temperature of 60 °C, and maximum acceptable difference between the melting temperatures of the left and right primes of 1 °C. Despite being quite restrictive and potentially reducing the number of primer pairs found, these temperature ranges were used to produce a set of primers suitable for multiplex PCR. All parameters that were not specified here were set to default values.

### Multiplex PCR and genotyping

For multiplex PCR, we used a methodology based on [29]. This approach uses, along with the reverse primers, forward primers modified by the addition of universal tails (Hill, Neo or M13; Table 1) and universal primers corresponding to the universal tails labeled with a fluorophore (Hill = FAM, Neo = Cal Fluorine Orange 560, and M13 = NED). Primers for the amplification of 26 tri- and tetra nucleotide loci were selected according to their product sizes and evaluated by PCR. We optimized the PCR conditions for each locus, by evaluating the duration of cycle steps, primer annealing temperature, reagent concentrations, and DNA concentration. Subsequently, 15 microsatellite loci with the best quality and performance results were combined according to their fragment size into two multiplex PCR reactions with nine and six loci in each reaction (9-plex and 6-plex, respectively). Each multiplex was initially tested on eight *G. agilis* samples to confirm the effectiveness of the multiplex PCR reaction for detecting the polymorphism previously found during the individual locus standardization process. After that, PCRs were performed on the remaining 22 individuals. Multiplex PCRs were performed in 15 µL final volume reactions with approximately 25 ng DNA, 1X Multiplex PCR Master Mix (Quiagen), 0.2 µM of each reverse primer, 0.05 µM of each modified forward primer, and 0.2 µM per locus of each universal primer, according to the tail present in the forward primers used in the multiplex. The conditions used in thermal cycling for the PCR reactions were as follows: initial denaturation at 95 °C for 15 min; 30 cycles at 94 °C for 30 s, 60 °C for 90 s, and 72 °C for 60 s; followed by 8 cycles at 94 °C for 30 s, 53 °C for 90 s, and 72 °C for 60 s; and a final extension step at 72 °C for 30 min. The quality of the amplified products was verified by 1% agarose gel electrophoresis stained with ethidium bromide. An aliquot of 2 µL of the PCR product was added to 7.5 µL of formamide and 0.5 µL of GeneScan 500 ROX size standard (Applied Biosystems), which was then denatured at 95 °C for 5 min. Denatured products were subjected to capillary electrophoresis using an ABI PRISM 3130 DNA sequencer (Applied Biosystems). Allele sizes were determined using the Microsatellite Plugin in the GENEIOUS 6.1.8 program (Biomatters).

**Table 1:** Primer sequences, motifs, tails, and multiplex combinations of the 15 microsatellite loci described for *Gracilinanus agilis*

Locus	NCBI accession	Primer (5’- 3’)	Motif	Tail^a^	Multiplex
Gag 14	PP786193	F: GATGGAGTTTCAACATTAGGGC	(AGAT)17	Hill	1
		R: TCCCTTAGCCTTAGTTTCTCCC			
Gag 19	PP786194	F: CAAACAATAGCTGCTGAAGCC	(ATCT)15	Neo	1
		R: TTTCCTTCCTCAAATATCCACC			
Gag 21	PP786195	F: TGACTTAAAAGGCTATCCACTGC	(GTT)14	M13	1
		R: GTGGCTCAGCAAGTTGTGG			
Gag 93	PP786196	F: TGATGTTAAACAGTGTGAGTGGC	(TCTA)14	Neo	1
		R: TGATCACCTAGGATTTGCTCG			
Gag 95	PP786197	F: TCATACAGCTAGTCAGTACCAGAGC	(GAT)14	Hill	1
		R: GGCTTACACTTGGGTAGGAGG			
Gag 96	PP786198	F: CCCTGTCATCCCTACAGTGC	(AGG)11	Hill	2
		R: GCTTCCGCAGACTAACTTGG			
Gag 115	PP786199	F: TTGACCCTTACAAATACTCTGGG	(ATT)14	M13	1
		R: AGAGAACTGAGCAGGCTTGG			
Gag 119	PP786200	F: TCTGAACCCAGAAAAGCTCC	(ATC)13	Neo	2
		R: GGGAGAGACTTGAGCCACC			
Gag 122	PP786201	F: AGCTTAGATCAATTCCTGCCC	(AATG)11	M13	2
		R: GGCAAAGGAGAAATCTAAAGGC			
Gag 124	PP786202	F: CGACCATATTCCTCCAGGC	(ACC)7	Hill	2
		R: CATCTCCTTGAACAGGTCCC			
Gag 155	PP786203	F: ACAAAGGCATTGGAATTTGG	(TAGA)15	Neo	2
		R: TTGTATACTGAAATTCATGTTTGGG			
Gag 181	PP786204	F: TGTAGGCTGAACTATTTGAGATGG	(AAC)11	Hill	1
		R: GAAGGAGGCCACTGTTTGG			
Gag 189	PP786205	F: GCTGCACCATTAGAGACTACTGC	(TAGA)12	Neo	1
		R: TGCCTCCCTTCATATTGTCC			
Gag 192	PP786206	F: AACCCTTGATTATGGTGACTCC	(CCAT)10	M13	1
		R: ACTGCCAGATAAAATTTCCCC			
Gag 197	PP786207	F: CACAAAAGACAATGGAGAGGC	(GAT)17	Hill	2
		R: GCGGGAACATAGAATGAAGG			

^a^Universal tail sequences added to the 5’ end of the forward primers: Hill (TGACCGGCAGCAAAATTG), Neomycin_rev (AGGTGAGATGACAGGAGATC), and M13(-21) (TGTAAAACGACGGCCAGT)

### Analysis and characterization of microsatellite loci

The number of alleles and observed and expected heterozygosity for each locus were estimated using FSTAT [30] and IDENTITY 4 [31]. Linkage disequilibrium between all loci pairs was tested using the log-likelihood ratio statistic (G-test) in GENEPOP 4.3 [32]. Deviations from the Hardy-Weinberg equilibrium (*HWE*) were evaluated using the exact-test [33] of the same program. The significance of the aforementioned analyses was assessed using 10,000 permutations with 5,000 batches and 500 iterations per batch. We checked for genotyping errors using MICROCHECKER [34] with 1000 Monte Carlo iterations. For all analyses, *p*-values were adjusted for multiple testing using the Bonferroni procedure [35]. The frequency of null alleles was estimated using the EM algorithm [36], as implemented in GENEPOP 4.3. Probabilities of paternity exclusion and genetic identity were estimated using IDENTITY 4.

## Results and discussion

### Mitogenome

**Fig. 1:**
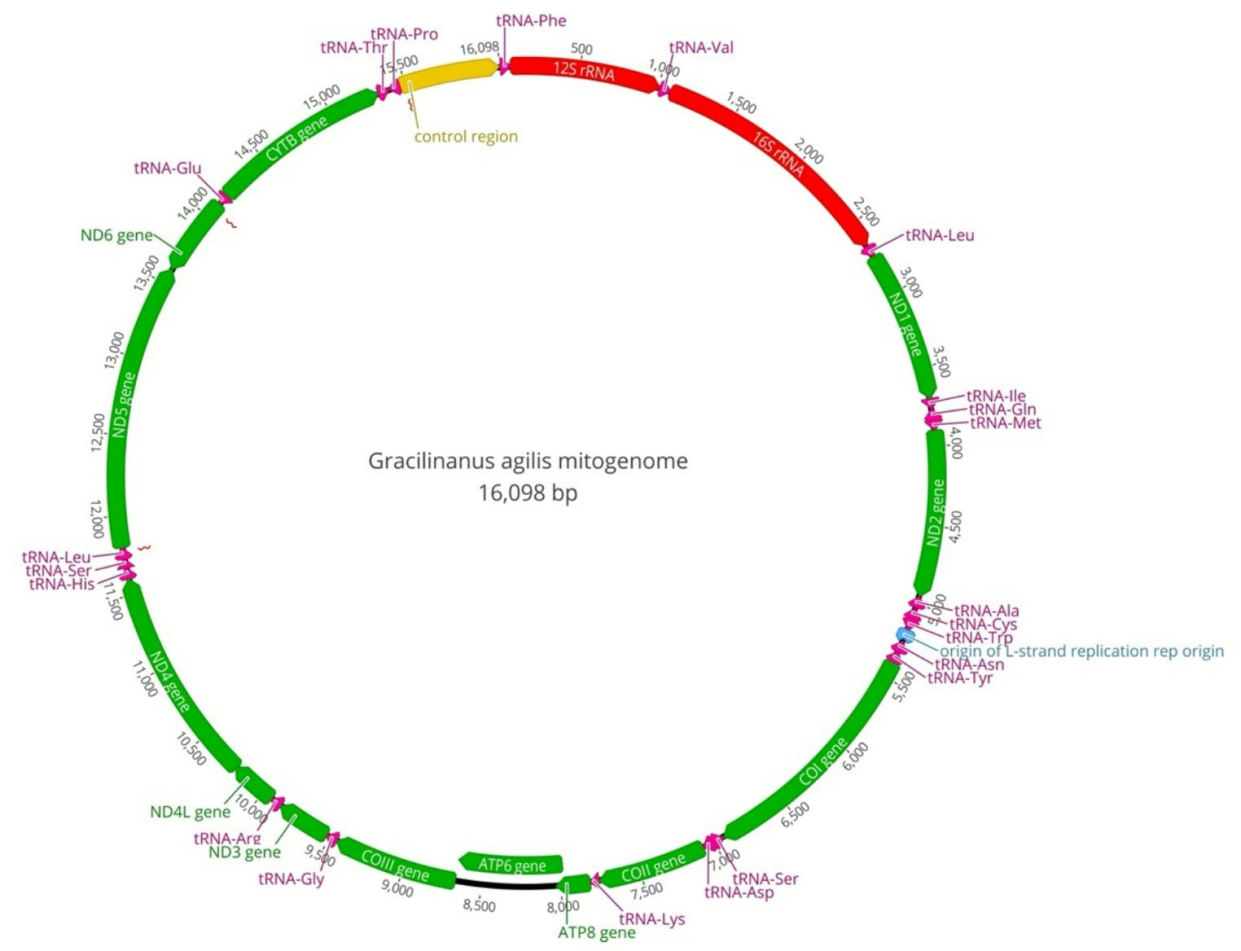
Mitogenome of *Gracilinanus agilis* (NC). Code genes are shown in green: COI-III indicates cytochrome c oxidase subunits 1–3; CYTB, cytochrome b; ATP6–8, ATPase subunits 6 and 8; ND1–6/4L, NADH dehydrogenase subunits 1–6/4 L. Transfer RNA genes are designated by three-letter abbreviation of the amino acid and shown in pink, ribosomal RNA genes (12 S and 16 S) are shown in red and control region is shown in orange. Genes encoded on the Heavy strand (H-strand) are represented by arrows oriented clockwise, whereas genes encoded on the Light strand (L-strand) are represented by arrows oriented counterclockwise

A total of 1,434 reads were mapped to the reference genome (NC_054268), with an average coverage of 14.8 x. The recovered *G. agilis* mitogenome (GenBank accession number PP831685) had 16,098 bp. The gene order and organization of the *G. agilis* mitogenome conformed to those of previously described marsupial genomes [37] (Fig. 1), comprising 22 tRNA genes, 2 rRNA genes, 13 protein-coding genes, and a 606 bp control region (D-loop). The mitogenome also included a noncanonical but possibly functional tRNA-Lys located between the COII and ATP8 genes.

Based on the reference mitogenome, only a short sequence (40 bp) of the ND6 gene was not sufficient covered by reads (< 2x), and these sites were filled with “Ns” in the assembled *G. agilis* mitogenome. The absence of coverage in this region is most likely attributable to natural intraspecific variation, which may reduce mapping efficiency in highly variable regions and consequently decrease read recovery during the mapping process. Overall, the mitogenome described here showed about 2% pairwise difference compared to the *G. agilis* reference genome.

### Microsatellites

In total, 2,331,566 sequences were obtained through Illumina HiSeq sequencing, of which 1,941,907 (83,29%) were retained after the first screening step. After filtering with the Galaxy Palfider platform, we found 4,199 microsatellite regions, resulting in 207 loci for which it was possible to design primers for PCR amplification (94 tri, 98 tetra, 11 penta, and 4 hexanucleotides). Of the 26 selected loci, 15 (58%) were successfully amplified, with PCR products within the expected allelic size range (Table 1).

All 15 loci were polymorphic. The mean number of alleles per locus was 7.73, and the number of alleles per locus varied from 2 to 15. The mean observed heterozygosity was 0.63, ranging from 0.17 to 0.97, whereas the mean expected heterozygosity was 0.72, ranging from 0.48 to 0.91 (Table 2). Linkage disequilibrium was not found for any pairwise comparison after Bonferroni correction (*p* < 0.000476).

**Table 2:** Characterization of 15 microsatellite loci for *Gracilinanus agilis*: sample size (N), number of alleles, allele size range (Min-Max), observed heterozygosity (H_O_), expected heterozygosity (H_E_), frequency of null alleles (F_NA_), probability of exclusion of paternity (P_EP_), and probability of identity (P_I_)

Locus	*N*	Alleles	Min‒Max	H_O_	H_E_	F_NA_	*P*_EP_^**^	*P*_I_^***^
Gag 14	30	9	212‒248	0.60	0.77	0.09	0.546	0.095
Gag 19	30	10	212‒260	0.67	0.80	0.12	0.603	0.071
Gag 21	28	10	227‒281	0.64	0.65	0.04	0.453	0.149
Gag 93	30	9	278‒314	0.90	0.85	0	0.675	0.047
Gag 95	30	8	290‒314	0.77	0.80	0.13	0.605	0.069
Gag 96	23	8	284‒308	0.30	0.79^*^	0.35	0.591	0.075
Gag 115	30	15	300‒342	0.83	0.93	0.16	0.827	0.014
Gag 119	30	7	287‒317	0.93	0.78	0.07	0.557	0.088
Gag 122	30	6	311‒331	0.77	0.75	0	0.504	0.113
Gag 124	30	2	316‒319	0.03	0.03	0.98	0.016	0.936
Gag 155	29	11	343‒383	0.97	0.87	0.02	0.720	0.035
Gag 181	30	5	364‒379	0.17	0.49^*^	0.26	0.261	0.316
Gag 189	29	5	388‒404	0.62	0.72	0.14	0.483	0.124
Gag 192	30	6	384‒404	0.43	0.79^*^	0.34	0.557	0.087
Gag 197	30	5	390‒405	0.80	0.78	0.11	0.548	0.091

^*^Departure from Hardy-Weinberg equilibrium after Bonferroni correction (*p* < 0.0033), ^**^P_EP_ (all loci combined) = 0.999, ^***^P_I_ (all loci combined) = 3.45 × 10^− 16^

Microchecker results indicated the presence of null alleles at four loci (Gag 14, Gag 96, Gag 181 and Gag 192), with estimated frequencies exceeding 20% for three of them (Table 2). Moreover, possible stuttering artifacts were detected at the Gag 181 locus. The exact test results indicated that three loci (Gag 96, Gag 181, and Gag 192) showed statistically significant departures from HWE after Bonferroni correction (*p* < 0.0033; Table 2). The observed deviations from HWE at these loci may be attributed to the presence of null alleles; however, the influence of population subdivision, as evidenced by the Wahlund effect [38], cannot be disregarded, given that the samples were collected from two distinct points (referred to as JB1 and JB2) situated 6.4 km apart within the Brasília Botanical Garden. The probability of paternity exclusion and the probability of identity using all loci were 0.999 and 3.45 × 10^− 16^, respectively.

## Conclusion

The microsatellite loci presented here are the first to be described for the marsupial *G. agilis*. This new set of loci, together with the newly reported mitogenome sequence, will be important for evaluating the genetic diversity and population structure of this species in the future. Additionally, the high paternity exclusion probability and low probability of identity indicate the potential of this set of markers for the analysis of kinship, social structure, and mating systems of this species.

## Data Availability

DNA sequences generated in this study were deposited in the GenBank genetic sequence database and are available under accession numbers PP786193 to PP786207 (microsatellite loci) and NC054268 (mitogenome).

## References

[1] Wenne R (2023) Microsatellites as molecular markers with applications in exploitation and conservation of aquatic animal populations. Genes 14:808. 10.3390/genes14040808 37107566 10.3390/genes14040808 PMC10138012

[2] Yu J, Yu X, Bi W, Li Z, Zhou Y, Ma R, Feng F, Huang C, Gu J, Wu W, Lan G, Zhang L, Chen C, Xue F, Liu J (2025) Mitogenome diversity and phylogeny of Felidae species. *Diversity *17:634. 10.1007/s11033-026-11765-3

[3] Chen Y, Guo C, Zhou S, Xiang Z (2023) The mating system of Hymalayan marmots as inferred by microsatellite markers. Curr Zool 69:654–657. 10.1093/cz/zoac079 37876642 10.1093/cz/zoac079 PMC10591142

[4] Meléndez-Rosa J, Lacey EA (2023) Monogamy or monogamish? Re-examining monogamy in *Peromyscus californicus*. Therya 14(2):207–214. 10.12933/therya-23-2241

[5] Astúa D, Cherem JJ, Teta P (2022) Taxonomic checklist of living American marsupials. In: Cáceres NC, Dickman CR (eds) American and Australasian Marsupials. Springer, Cham, pp 1–48. 10.10007/978-3-030-88800-8_31-1

[6] Dias IMG, Amato G, Cunha HM, DeSalle R, Paglia AP, Peterson JK, Fonseca CG (2009) Isolation, characterization and cross-species amplification of new microsatellite markers for three opossum species of the Didelphidae family. Conserv Genet Resour 1:405–410. 10.1007/s12686-009-9094-8

[7] Sommer S, Schmidt A, Fernandez F, Püttker T, Pardini R (2009) Development and characterization of microsatellite loci in the marsupial *Marmosops incanus* (Lund, 1840) of the Brazilian Atlantic rain forest using genome screening and restriction ligation. Mol Ecol Resour 9:1460–1466. 10.1111/j.1755-0998.2009.02759.x 21564933 10.1111/j.1755-0998.2009.02759.x

[8] Valladares-Gómez A, Celis-Diez JL, Sepúlveda-Rodríguez C, Inostroza-Michael O, Hernández CE, Eduardo Palma R (2019) Genetic diversity, population structure, and migration scenarios of the marsupial Monito del Monte in south-central Chile. J Hered 110(6):651–661. 10.1093/jhered/esz049 31420661 10.1093/jhered/esz049

[9] Creighton GK, Gardner AL (2008) Genus *Gracilinanus* Gardner and Creighton, 1989, pp 43–50. In: Gardner AL (ed) Mammals of South America, Vol 1: Marsupials, Xenarthrans, Shrews and Bats. University of Chicago Press, Chicago, IL, USA

[10] Camargo NF, Ribeiro JF, de Camargo AJA, Vieira EM (2013) Diet of gracile mouse opossum *Gracilinanus agilis* (Didelphimorphia:Didelphidae) in a neotropical savanna: intraspecific variation and resource selection. Acta Theriol 59:183–191. 10.1007/s13364-013-0152-y

[11] Camargo NF, dos Reis GG, Mendonça AF, Laumann RA, Nardoto GB, Camargo AJA, Vieira EM (2022) Native marsupial acts as an in situ biological control agent of the main soybean pest (*Euschistus heros*) in the Neotropics. Eur J Wildl Res 68(62). 10.1007/s10344-022-01609-3

[12] Camargo NF, Cruz RMS, Ribeiro JF, Vieira EM (2011) Frugivoria e potencial dispersão de sementes pelo marsupial *Gracilinanus agilis* (Didelphidae: Didelphimorphia) em áreas de Cerrado no Brasil Central. Acta Bot Bras 25(3):646–656. 10.1590/S0102-33062011000300018

[13] Souza DP, Asfora PH, Lira TC, Astúa D (2010) Small mammals in Barn Owl (*Tyto alba* – Aves, Strigiformes) pellets from Northeastern Brazil, with new records of *Gracilinanus* and *Cryptonanus* (Didelphimorphia, Didelphida). Mamm Biol 75(4):370–374. 10.1016/j.mambio.2009.08.003

[14] Brandão EMV, Xavier SCC, Carvalhaes JG, D’Andrea PS, Lemos FG, Azevedo FC, Cássia-Pires R, Jansen AM, Roque ALR (2019) Trypanosomatids in Small Mammals of an Agroecosystem in Central Brazil: Another Piece in the Puzzle of Parasite Transmission in an Anthropogenic Landscape. Pathogens 8(4):190. 10.3390/pathogens8040190 31615153 10.3390/pathogens8040190 PMC6963188

[15] Carmignotto AP, Solari S, de la Sancha N, Costa L (2015) *Gracilinanus agilis*. The IUCN Red List of Threatened Species 2015:e.T9417A22169828. 10.2305/IUCN.UK.2015-4.RLTS.T9417A22169828.en

[16] Colli GR, Vieira CR, Dianese JC (2020) Biodiversity and conservation of the Cerrado: recent advances and old challenges. Biodivers Conserv 29(5):1465–1475. 10.1007/s10531-020-01967-x

[17] Lopes GP, Leiner NO (2015) Semelparity in a population of *Gracilinanus agilis* (Didelphimorphia: Didelphidae) inhabiting the Brazilian cerrado. Mamm Biol 80(1):1–6. 10.1016/j.mambio.2014.08.004

[18] Puida DBC, Paglia AP (2015) Primary productivity and demography of *Gracilinanus agilis*, a small semelparous marsupial. J Mammal 96(1):221–229. 10.1093/jmammal/gyu030

[19] Zangrandi PL, Vieira EM (2023) Semelparous Reproductive Strategy in New World Marsupials. In: Cáceres NC, Dickman CR (eds) American and Australasian Marsupials. Springer, Cham. 10.1007/978-3-031-08419-5_19

[20] Leiner NO, Setz EZF, Silva WR (2008) Semelparity and factors affecting the reproductive activity of the Brazilian slender opossum (*Marmosops paulensis*) in southeastern Brazil. J Mammal 89(1):153–158. 10.1644/07-MAMM-A-083.1

[21] Baladrón AV, Malizia AI, Bó MS, Liébana MS, Bechard MJ (2012) Population dynamics of the southern short-tailed opossum (*Monodelphis dimidiate*) in the Pampas of Argentina. Aust J Zool 60(4):238–245. 10.1071/ZO12037

[22] Sambrook J, Russell DW (2006) The condensed protocols from molecular cloning: a laboratory manual. Cold Spring Harbor Laboratory Press, Cold Spring Harbor, New York

[23] Bernt M, Donath A, Jühling F, Externbrink F, Florentz C, Fritzsch G, Pütz J, Middendorf M, Stadler PF (2013) MITOS: improved de novo metazoan mitochondrial genome annotation. Mol Phylogenet Evol 69(2):313–319. 10.1016/j.ympev.2012.08.023 22982435 10.1016/j.ympev.2012.08.023

[24] Griffiths SM, Fox G, Briggs PJ, Donaldson IJ, Hood S, Richardson P, Leaver GW, Truelove N, Preziosi RF (2016) A Galaxy-based bioinformatics pipeline for optimized, streamlined microsatellite development from Illumina next-generation sequencing data. Cons Genet Resour 8(4):481–486. 10.1007/s12686-016-0570-7 10.1007/s12686-016-0570-7 PMC7175698 32355508

[25] Andrews S, FastQC: a quality control tool for high throughput sequence data (Online). Available at: https://www.bioinformatics.babraham.ac.uk/projects/fastqc/

[26] Bolger AM, Lohse M, Usadel B (2014) Trimmomatic: a flexible trimmer for Illumina sequence data. Bioinformatics 30(15):2114–2120. 10.1093/bioinformatics/btu170 24695404 10.1093/bioinformatics/btu170 PMC4103590

[27] Castoe TA, Poole AW, de Koning J, Jones KL, Tomback DF, Oyler-McCance SJ, Fike JA, Lance SL, Streicher JW, Smith EN, Pollock DD (2012) Rapid microsatellite identification from Illumina paired-end genomic sequencing in two birds and a snake. PLoS ONE 7(2):e3095. 10.1371/journal.pone.0030953 10.1371/journal.pone.0030953 PMC3279355 22348032

[28] Untergasser A, Cutcutache I, Koressaar T, Ye J, Faircloth BC, Remm M, Rozen SG (2012) Primer3 – new capabilities and interfaces. Nucleic Acids Res 40:e115 22730293 10.1093/nar/gks596 PMC3424584

[29] Vartia S, Collins PC, Cross TF, FitzGerald RD, Gauthier DT, McGinnity P, Mirimin L, Carlsson J (2014) Multiplexing with three-primer PCR for rapid and economical microsatellite validation. Hereditas 151(2–3):43–54. 10.1111/hrd2.00044 25041267 10.1111/hrd2.00044

[30] Goudet J (2001) FSTAT. Ver. 2.9.3. A program to estimate and test gene diversities and fixation indices. http://www2.unil.ch/popgen/softwares/fstat

[31] Wagner HW, Sefc KM (1999) IDENTITY 1.0. Centre for Applied Genetics. University of Agricultural Sciences, Vienna

[32] Rousset F (2008) GENEPOP’007: a complete re-implementation of the GENEPOP software for Windows and Linux. Mol Ecol Resour 8(1):103–106. 10.1111/j.1471-8286.2007.01931 21585727 10.1111/j.1471-8286.2007.01931.x

[33] Haldane JBS (1954) An exact test for randomness of mating. J Genet 52(1):631–635

[34] Van Oosterhout C, Hutchinson WF, Wills DPM, Shipley P (2004) MicroChecker: software for identifying and correcting phenotyping errors in microsatellite data. Mol Ecol Notes 4(3):535–538. 10.1111/j.1471-8286.2004.00684.x

[35] Rice WR (1989) Analyzing tables of statistical tests. Evolution 43(1):223–225. 10.2307/2409177 28568501 10.1111/j.1558-5646.1989.tb04220.x

[36] Dempster AP, Laird NM, Rubin DB (1977) Maximum likelihood from incomplete data via the EM algorithm. J R Stat Soc Ser B (Stat Methodol) 39(1):1–22

[37] Nilson MA, Gullberg A, Spotorno AE, Arnason U, Janke A (2003) Radiation of extant marsupials after the K/T boundary: evidence from complete mitochondrial genomes. J Mol Evol 57 Suppl 1S3–12. 10.1007/s00239-003-0001-8 10.1007/s00239-003-0001-8 15008398

[38] Garnier-Géré PH, Chikhi L (2013) Population subdivision, Hardy–Weinberg equilibrium and the Wahlund effect. eLS. 10.1002/9780470015902.a0005446.pub3

